# Synergistic effects of putative Ca^2+^-binding sites of calmodulin in fungal development, temperature stress and virulence of *Aspergillus fumigatus*

**DOI:** 10.1080/21505594.2023.2290757

**Published:** 2023-12-12

**Authors:** Xingyue Li, Ruoyun Feng, Pan Luo, Yuanwei Zhang, Ling Lu

**Affiliations:** Jiangsu Key Laboratory for Microbes and Functional Genomics, Jiangsu Engineering and Technology Research Center for Microbiology, College of Life Sciences, Nanjing Normal University, Nanjing, China

**Keywords:** Calcium homeostasis, calmodulin, EF-hand, virulence, *A. fumigatus*

## Abstract

In pathogenic fungi, calcium-calmodulin-dependent serine-threonine-specific phosphatase calcineurin is involved in morphogenesis and virulence. Therefore, calcineurin and its tightly related protein complexes are attractive antifungal drug targets. However, there is limited knowledge available on the relationship between *in vivo* Ca^2+^-binding sites of calmodulin (CaM) and its functions in regulating stress responses, morphogenesis, and pathogenesis. In the current study, we demonstrated that calmodulin is required for hyphal growth, conidiation, and virulence in the human fungal pathogen, *Aspergillus fumigatus*. Site-directed mutations of calmodulin revealed that a single Ca^2+^-binding site mutation had no significant effect on *A. fumigatus* hyphal development, but multiple Ca^2+^-binding site mutations exhibited synergistic effects, especially when cultured at 42 °C, indicating that calmodulin function in response to temperature stress depends on its Ca^2+^-binding sites. Western blotting implied that mutations in Ca^2+^-binding sites caused highly degraded calmodulin fragments, suggesting that the loss of Ca^2+^-binding sites results in reduced protein stability. Moreover, normal intracellular calcium homeostasis and the nuclear translocation of the transcriptional factor CrzA are dependent on Ca^2+^-binding sites of *Af*CaM, demonstrating that Ca^2+^-binding sites of calmodulin are required for calcium signalling and its major transcription factor CrzA. Importantly, *in situ* mutations for four Ca^2+^-binding sites of calmodulin resulted in an almost complete loss of virulence in the *Galleria mellonella* wax moth model. This study shed more light on the functional characterization of putative calcium-binding sites of calmodulin in the morphogenesis and virulence of *A. fumigatus*, which enhances our understanding of calmodulin biological functions in cells of opportunistic fungal pathogens.

## Introduction

As an intracellular secondary messenger in eukaryotic cells, Ca^2+^ plays a crucial role in regulating various cellular processes, including secretion, synaptic transmission, and cytokinesis [1,2]. The intracellular Ca^2+^ concentration is strictly and precisely controlled by a sophisticated calcium homeostasis system, which consists of various calcium channels, calcium pumps, and calcium antiporters [2–4]. Calmodulin (CaM), a Ca^2+^-binding protein with predicted 3–4 EF-hands to bind calcium, interacts with many other proteins in the cell, and acts as a signalling molecule in a wide variety of cellular functions [5–7]. In humans, CaM is encoded by three independent genes, is a 148 amino acid, highly conserved calcium sensor protein that contains four EF-hand Ca^2+^-binding motifs [8]. In the model yeast *Schizosaccharomyces pombe, Saccharomyces cerevisiae* and the filamentous fungus *Aspergillus nidulans*, CaM is encoded by a single gene, and Ca^2+^ binding to CaM is required for activation in *S. pombe* and *A. nidulans*. However, only the Ca^2+^-independent functions of CaM are essential for growth in the budding yeast *S. cerevisiae*. CaM from *S. cerevisiae* binds only three Ca^2+^ ions, with the fourth EF-hand motif being not required for binding calcium [5], whereas CaM from vertebrate systems binds four Ca^2+^ ions with each EF-hand motif [9]. Although CaM is necessary for the development of budding yeast, high-affinity Ca^2+^ binding to CaM is not necessary because the yeast can thrive when CaM is mutated [10]. In contrast to *S. cerevisiae*, high-affinity Ca^2+^ binding to CaM is required to support the growth of *S. pombe* and *A. nidulans* [6,11]. Crz1/CrzA, a calcineurin-responsive zinc finger 1 transcription factor, is the most well-studied downstream target of calcineurin [12–15]. It has been demonstrated that once the concentration of intracellular calcium increases, CaM binds to calcium and activates calcineurin, leading to the dephosphorylation of Crz1 entering the nucleus in the cell [14,15]. Crz1 is involved in many biological processes in fungi, including hyphal growth, cell wall integrity, pathogenicity and stress response [16–18]. Although great progress has been made in understanding CaM in model fungi, especially yeast, it provides an excellent roadmap but cannot substitute for studies on fungal pathogens. Fungal infections are becoming a very important concern for human health worldwide because an increasing number of immunocompromised patients are susceptible to such infections with a high mortality rate [19]. Importantly, many lines of evidence have demonstrated that calcium signalling is involved in fungal virulence and antifungal resistance [7,13,20–22]. Calcium signalling via CaM may be critical for stress responses in two non-pathogenic fungi, *S. pombe* and *Aspergillus oryzae* [23,24]. In the plant pathogenic fungi *Colletotrichum trifolii* and *Pyricularia grisea*, the development of a specialized infectious structure called the appressorium can be inhibited by the expression of CaM antisense RNA (*C. trifolii*) or by CaM antagonists and calcium chelators (*M. grisea*) [25,26]. Moreover, the *cam* gene from the human pathogen *Paracoccidioides brasiliensis* was investigated, and experiments using CaM antagonists revealed its role in the mycelial-to-yeast transition, which is essential for infection [27]. Additionally, a *Cryptococcus neoformans* strain with a mutation in the *cam* gene (*CaM1*) has been obtained *in vitro*, which caused a marked reduction in CaM expression, leading to a growth defect at 37 °C [28]. However, the role of CaM in the human pathogen *Aspergillus fumigatus* remains elusive.

Among species within the *Aspergillus* genus, *A. fumigatus* is the most ubiquitous aetiological factor of aspergillosis. Such evolutionary success is related to (1) small conidia size allows penetration to the lower respiratory tract system and escaping clearance by mucociliary forces; (2) the presence of melanin in the cell wall enables withstanding reactive oxygen species and phagocytosis; (3) abundance of negatively charged sialic acid on the surface permits *A. fumigatus* to effectively bind to the basal lamina proteins once inside the host lung [29,30]. More than 200,000 cases of invasive aspergillosis are thought to occur annually, with a 40–100% mortality rate [30–32]. Furthermore, intrinsic and acquired resistance to antifungals in *A. fumigatus* results in reduced drug efficacy [33]. To date, little is known about the function of *A. fumigatus* CaM. In this study, the functions of the four predicted *A. fumigatus* Ca^2+^-binding sites of CaM were verified. We discovered that there were no detectable differences in colony morphology between different single Ca^2+^-binding site mutants compared to their parental wild type, respectively, but mutations in multiple Ca^2+^-binding sites resulted in synergistic destructive effects. Ca^2+^-binding sites are especially required for *A. fumigatus* growth when cultured at 42 °C, implying that calcium binding to CaM is essential for fungal survival to deal with temperature stress. Notably, our study found that mutation of the Ca^2+^-binding sites in CaM results in reduced virulence. Collectively, this study provides direct evidence that Ca^2+^ binding-dependent CaM plays important roles in *A. fumigatus* development, temperature stress, and virulence performed *in vivo* on the model of *Galleria mellonella*.

## Results

### *Af*CaMis required for normal hyphal growth and conidiation

Based on a NCBI BLASTp search using the protein sequence of *S. cerevisiae* CaM as the query, we identified the predicted CaM in *A. fumigatus*, *Af*CaM (AFUB_067160). The predicted *Af*CaM open reading frame (ORF) includes 450 bp nucleotides with six introns and encodes a protein 149 amino acids in length. *Af*CaM is highly conserved as shown by its high identity with other fungal homologs such as *A. nidulans* (100%), *S. cerevisiae* (60%), and *S. pombe* (69%) (Fig. S1).

**Figure 1.**
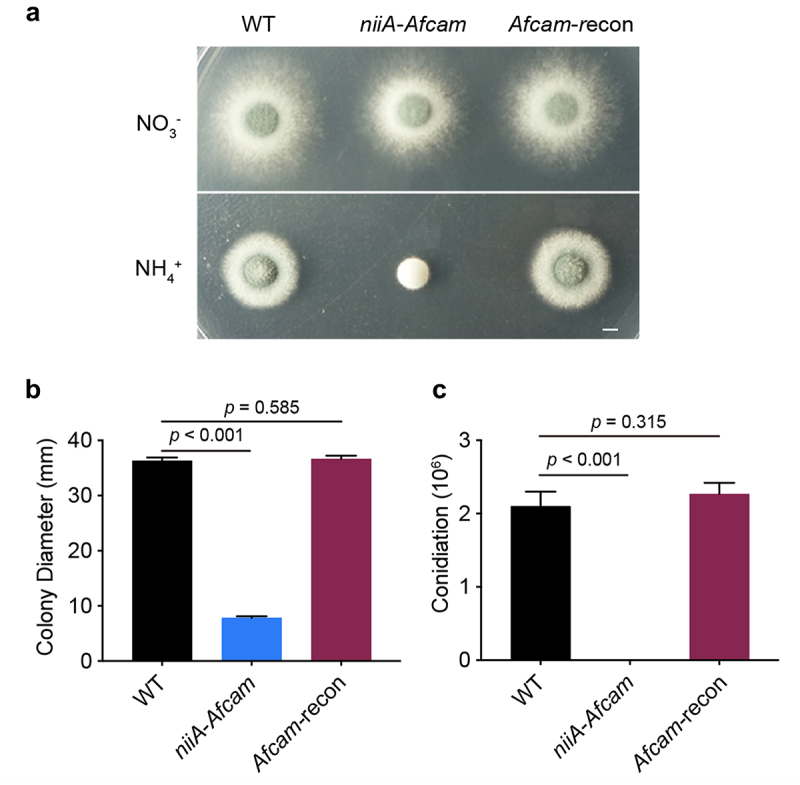
CaM is essential for *A. fumigatus* hyphal growth and conidiation. (a) colony morphology of the indicated *cam* strains on the inducing or repressing medium at 37 °C for 2.5 days. Bar, 0.5 cm. (b) relative quantitative analysis for the colony diameter and (c) conidiation of the WT, *niiA-Afcam* and *Afcam*-recon strains under NO_3_^−^ for *niiA*-induced condition and NH_4_^+^ for *niiA*-repressed condition. The data are presented as the mean ± standard deviation of three independent experiments. Statistical analysis was performed by Student’s t-test.

To explore the biological functions of CaM in *A. fumigatus*, we attempted to construct the full-length deletion strain *Afcam* many times using a homologous recombination strategy but were unsuccessful. This may be due to the essential role of CaM in *A. fumigatus* viability. Therefore, a conditional *niiA-Afcam* strain was generated, which conditionally induced *Afcam* under a nitrogen source of NO_3_^−^ and repressed *cam* under NH_4_^+^ conditions. The correct construction was confirmed by diagnostic PCR analysis (Fig. S2). As shown in Figure 1a, the conditional *niiA-Afcam* strain displayed normal hyphal growth and conidiation on the inducing medium. Under repressing conditions, it exhibited no conidiation or hyphal growth when compared with wild type (WT) (Figure 1a). To verify that these defects are specifically caused by *Afcam* repression, we constructed an *Afcam*-complemented strain (*Afcam*-recon) by reintroducing a parental WT *cam* gene into conditional *niiA-Afcam* strain, allowing for genetic complementation when cultured on the repressing medium. Among transformants, the one that was able to show a comparable colony growth phenotype compared to that of the WT strain under repressing conditions was picked (Figure 1a), suggesting that the complemented *cam* gene can rescue the growth defects when cultured on *cam* repression conditions in *A. fumigatus*. The relative quantitative analysis to Figure 1a for colony growth and conidiation is shown in Figure 1b. These results suggest that *cam* is required for hyphal growth and conidiation in *A. fumigatus*.

### Four predicted Ca^2+^-binding EF-hands within CaM exert distinct functions in *A.* *fumigatus*

**Figure 2.**
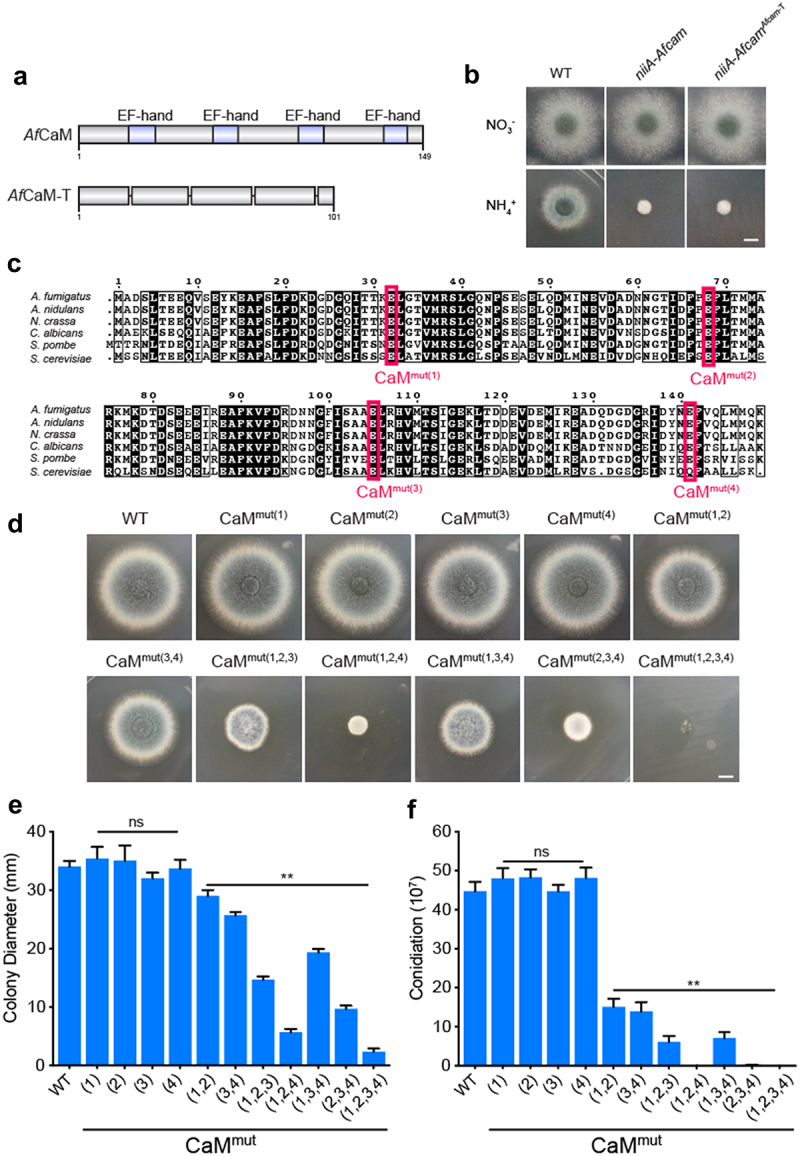
Four Ca^2+^-binding sites of *A. fumigatus* CaM exhibit different functions. (a) schematic diagram of the *Af*CaM and *Af*CaM-T. The *Afcam-T* gene was transferred to the conditional *niiA-Afcam* strain when cultured on the repressing medium. (b) colony morphology of the truncated mutant on YUU solid medium at 37 °C for 2.5 days. Bar, 0.5 cm. (c) the mutations in EF-loops of *Af*CaM. (d) colony morphology of the indicated *cam* mutants grown on YUU solid medium at 37 °C for 2.5 days. Bar, 0.5 cm. The residue numbering for *Af*CaM is based on the conserved CaM coding sequence. For example, CaM^mut(1,2)^ is the simultaneous mutation of residues 1 and 2 of *Af*CaM Ca^2+^-binding sites. (e) relative quantitative analysis of the radial growth and (f) conidiation of the WT and *cam* mutants assessed after 2.5 days. The data are presented as the mean ± standard deviation of three independent experiments. Statistical analysis was performed using one-way ANOVA with multiple comparison tests. **p < 0.01; ns, not significant.

To dissect the structure of CaM, we used SMART protein analysis, which revealed that *Af*CaM contains four EF-hand motifs (Figure 2a). Each EF-hand provides an acidic loop, or “EF-loop,” for calcium coordination [34,35]. It has been reported that EF hands are required for calcium binding and CaM conformational changes [36,37]. To further explore the importance of four predicted Ca^2+^-binding loops for CaM function in *A. fumigatus*, we constructed the truncated mutant *niiA-Afcam*^*Afcam-T*^ which removed the predicted EF-hand sequence encoding amino acids–21–32、57–68、94–105 and 130–141 in *Af*CaM. Then the *Afcam-T* gene was transferred to the conditional *niiA-Afcam* strain when cultured on the repressing medium. Therefore, there is one copy of *niiA-Afcam* and an additional copy of *Afcam-T* in transformants, referred to as *niiA-Afcam*^*Afcam-T*^. The defective phenotype of *niiA-Afcam*^*Afcam-T*^ was similar to that of *niiA-Afcam* mutant under repressing conditions (Figure 2b), indicating that the lack of four EF-hands results in a complete loss of CaM functionality in *A. fumigatus*.

As the truncated mutant *niiA-Afcam*^*Afcam-T*^ may lead to instability or conformational changes in *Af*CaM protein, we further investigated the functions of the Ca^2+^-binding sites in each EF-hand. Since previous studies in yeast have reported that the last amino acid of the EF-hand plays a key role in binding to calcium [38], we constructed mutants in which the last conserved glutamic acid residue in each EF-hand was altered to alanine, while other residues remained unchanged by site-directed mutagenesis. These single mutations of E32A, E68A, E105A, and E141A in EF-loops are referred to as CaM^mut(1)^, CaM^mut(2)^, CaM^mut(3)^, and CaM^mut(4)^ based on the order of the four EF-hands (Figure 2c). As shown in Figure 2D, strains that embedded the individual mutations (CaM^mut(1)^, CaM^mut(2)^, CaM^mut(3)^, CaM^mut(4)^) did not show any detectable differences compared to WT, whereas the double mutations CaM^mut(1,2)^ and CaM^mut(3,4)^ exhibited slight growth defects. Notably, the simultaneous mutations of residues 2 and 4 in *Af*CaM resulted in nearly no grown colonies of *A. fumigatus* (Figure 2d), implying that these two residues have key roles for colony growth and loss of both would significantly impair CaM function. In comparison, strains carrying CaM^mut(1,2,3)^ and CaM^mut(1,3,4)^ mutations showed less severe growth defects (Figure 2d). However, mutations in four Ca^2+^-binding sites of CaM^mut(1,2,3,4)^ resulted in a serious defective phenotype of *A. fumigatus* (Figure 2d), suggesting that these combined four predicted Ca^2+^-binding sites of CaM are essential for *A. fumigatus* growth. Taken together, these data reveal that a single Ca^2+^-binding site mutation in *Af*CaM had no obvious influence on *A. fumigatus* hyphal development and conidiation, whereas multiple Ca^2+^-binding site mutations exhibited synergistic lethal effects.

### Putative Ca^2+^-binding sites are important for CaM stabilization

**Figure 3.**
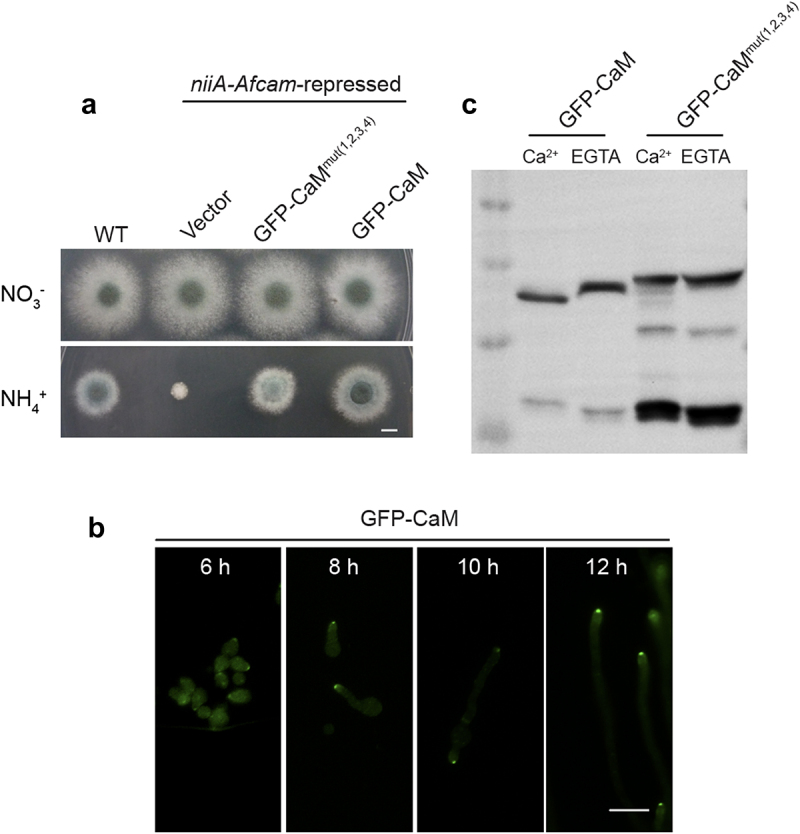
Four Ca^2+^-binding sites of CaM are required for its stabilization. (a) colony morphology of the WT、*niiA-Afcam*、*niiA*-CaM^GFP-CaM(1,2,3,4)^ and *niiA*-CaM^GFP-^^CaM^ strains on the inducing and repressing medium at 37 °C for 2.5 days. Bar, 0.5 cm. (b) localization of GFP-CaM strain at 6 h, 8 h, 10 h and 12 h, respectively. Bar, 5 μm. (c) western blotting analysis of the *Af*CaM conformational changes in the GFP-CaM and GFP-CaM^mut(1,2,3,4)^ strains under 1 mM CaCl_2_ or 5 mM EGTA.

To gain insight into the subcellular localization of CaM in *A. fumigatus*, we used *niiA-Afcam* as the background strain to construct *niiA-Afcam*^GFP-^^CaM^ and *niiA-Afcam*^GFP-^^CaM^^(1,2,3,4)^ which expressed *Afcam* and *Afcam*^(1,2,3,4)^ fused with the N-terminal green fluorescent protein (GFP) under the control of its native promoter. Then we compared colony phenotypes of *niiA-Afcam*^GFP-^^CaM^ and *niiA-Afcam*^GFP-^^CaM^^(1,2,3,4)^ with the parental conditional strain under repressing conditions. Among transformants, the *niiA-AfCaM*^GFP-^^CaM^ strain exhibited similar phenotypes to that of the WT strain under either expressing or repressing conditions (Figure 3a), implying that fused GFP with CaM could not affect CaM normal function. However, the colony phenotype of *niiA-Afcam*^GFP-^^CaM^^(1,2,3,4)^ showed slight defects on the repressing medium (Figure 3a), suggesting that *niiA-Afcam*^GFP-^^CaM^ ^(1,2,3,4)^ was unable to rescue functions of repressed CaM. This implies that mutations in CaM four EF-hands would affect CaM normal functions.

Notably, there was a discrepancy in colony phenotypes between CaM^mut(1,2,3,4)^ in Figure 2d and *niiA-Afcam*^GFP-^^CaM^^(1,2,3,4)^ in Figure 3a. Because the former one was generated by site-directed mutagenesis, resulting in nearly no grown colonies of *A. fumigatus*. However, the *niiA-Afcam*^GFP-^^CaM^^(1,2,3,4)^ strain was constructed by reintroducing an *Afcam*^(1,2,3,4)^ gene into the *niiA-Afcam* strain and then examined its function under repressing conditions, in which the *niiA-Afcam* expression could not be fully turn-off on the repressing medium (Fig. S2).

Fluorescence microscopy revealed that GFP-CaM was concentrated at the hyphal apex (Figure 3b). In addition, the protein expression of GFP-CaM and GFP-CaM^mut(1,2,3,4)^ was detected using western blotting analysis. As shown in Figure 3c, GFP-CaM moved slightly faster by additional Ca^2+^ treatment compared to that without Ca^2+^ treatment, which reflects the conformational difference between the Ca^2+^-free and Ca^2+^-saturated forms of the protein. However, the mobilities of the mutant GFP-CaM^mut(1,2,3,4)^ were almost identical in the presence or absence of Ca^2+^ (Figure 3c), implying that the mutations of the four Ca^2+^-binding sites resulted in a deficiency of calcium binding to CaM. This provides indirect evidence that mutations in all four Ca^2+^-binding sites could, to a great extent, affect Ca^2+^ affinity. However, the expression levels of GFP-CaM^mut(1,2,3,4)^ were not significantly different from those of GFP-CaM (Figure 3c), indicating that the four Ca^2+^-binding mutations did not affect CaM expression. Notably, apart from the predicted mass of approximately 48 kDa (21.0 kDa of *Af*CaM plus 26.9 kDa of GFP), another band between 10 and 17 kDa was observed in the GFP- CaM^mut(1,2,3,4)^ strain, but not in the GFP-CaM strain (Figure 3c), which was likely to be part of the degraded GFP-CaM fusion protein fragments. These findings suggest that the loss of key Ca^2+^-binding sites of CaM results in its destabilization.

### The CaM function depends on its Ca^2+^-binding sites

**Figure 4.**
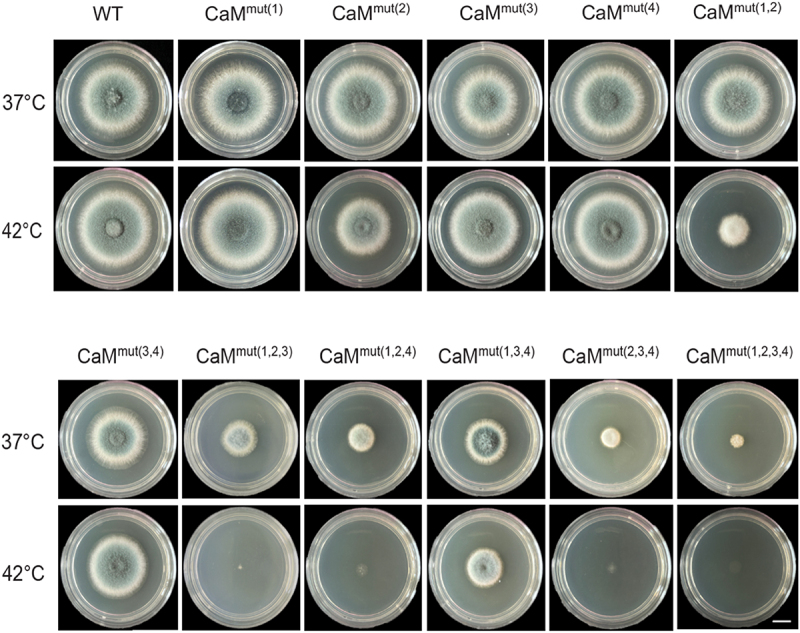
The CaM function depends on its Ca^2+^-binding sites. Colony morphology of the indicated *Afcam* mutants and parental wild type under high temperature. Strains were grown on YUU solid medium at 37 °C and 42 °C for 2 days. Bar, 0.5 cm.

Given that the activation of the calcium pathway through CaM plays an essential role in response to various environmental stresses [21,28], we assayed the phenotypes of relative *Afcam* mutants under high-temperature and osmotic stress. As shown in Figure 4, when grown at 42 °C on the rich medium (YUU), CaM^mut(1)^, CaM^mut(3)^ and CaM^mut(4)^ displayed similar phenotypes compared to those of WT. However, CaM^mut(3,4)^ and CaM^mut(1,3,4)^ exhibited relatively defective morphological phenotypes (Figure 4). Interestingly, all mutants embedding the putative second calcium-binding loop, CaM2, including CaM^mut(2)^, CaM^mut(1,2)^, CaM^mut(1,2,3)^, CaM^mut(1,2,4)^, CaM^mut(2,3,4)^ and CaM^mut(1,2,3,4)^ showed hypersensitive phenotypes at 42 °C to some content (Figure 4), suggesting that the second EF-hand plays a crucial role in adaptation to the heat stress. In comparison, sorbitol (1 M) did not cause significant changes in the colony phenotypes (Fig. S3). These data imply that the *Afcam* mutants that lacked Ca^2+^-binding sites would lead to the inability of *A. fumigatus* to grow at high temperatures. Collectively, these results indicate that CaM function in response to heat stress depends on Ca^2+^-binding sites.

### Nuclear accumulation of CrzA induced by calcium relies on the Ca^2+^-binding ability of CaM

**Figure 5.**
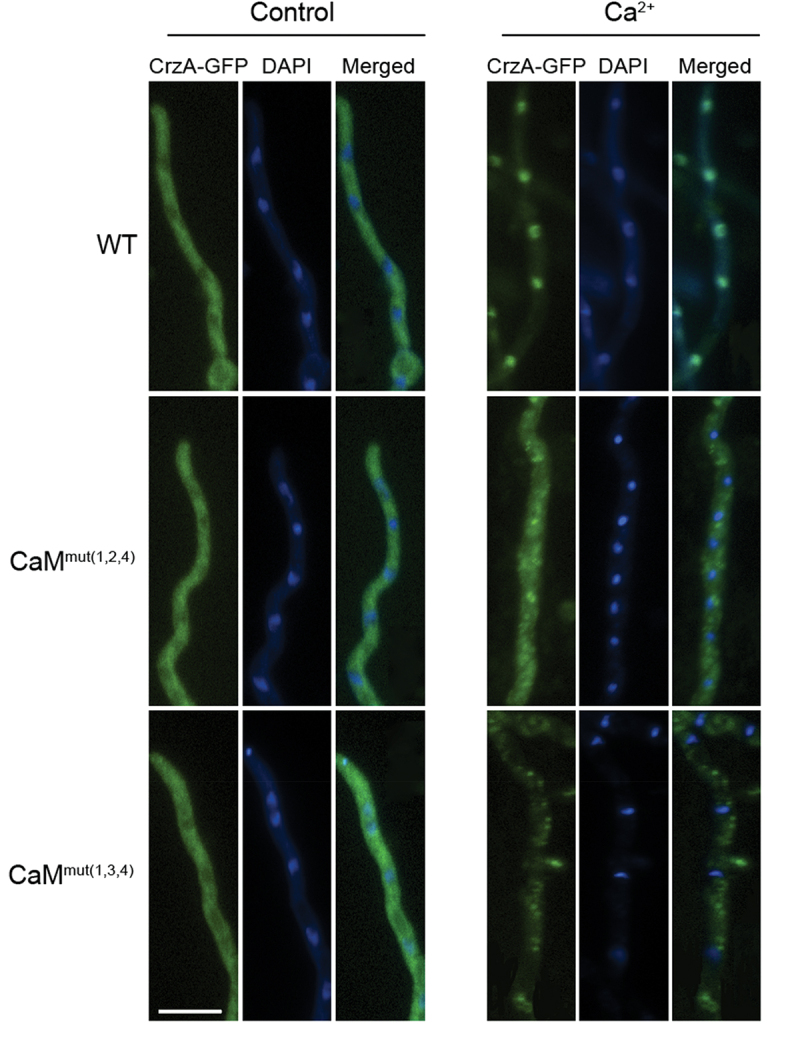
Localization of CrzA-GFP in three indicated strains (WT、CaM^mut(1,2,4)^、CaM^mut(1,3,4)^). Strains were grown in minimal media (MM) for 10 h and observed by fluorescence microscopy with or without stimulation of 100 mM CaCl_2_. The nuclear localization of cells was visualized by DAPI staining. Bar, 10 μm.

The transcriptional factor Crz1 is activated by the Ca^2+^/CaM/calcineurin pathway, and its nucleocytoplasmic trafficking requires fully functional calcineurin [7]. To determine whether the binding of calcium in CaM affects the localization of CrzA (Crz1 ortholog in *A. fumigatus*), we constructed C-terminal GFP-tagged CrzA strains in the background of WT, CaM^mut(1,2,4)^ and CaM^mut(1,3,4)^ to visualize the trafficking of CrzA. As shown in Figure 5, CrzA-GFP in the WT strain remained preferentially cytoplasmic in hyphal cells when cultured in normal minimal media (MM). As predicted, CrzA-GFP was predominantly induced by calcium (100 mM) from the cytosol to the nucleus (Figure 5). However, nucleocytoplasmic trafficking of CrzA did not occur in either CaM^mut(1,2,4)^ or CaM^mut(1,3,4)^ strains, suggesting that Ca^2+^-binding sites are required for the trans-localization of CrzA from the cytoplasm to the nuclei in *A. fumigatus*.

### Mutations in Ca^2+^-binding sites lead to the disorder of cytoplasmic calcium homeostasis

**Figure 6.**
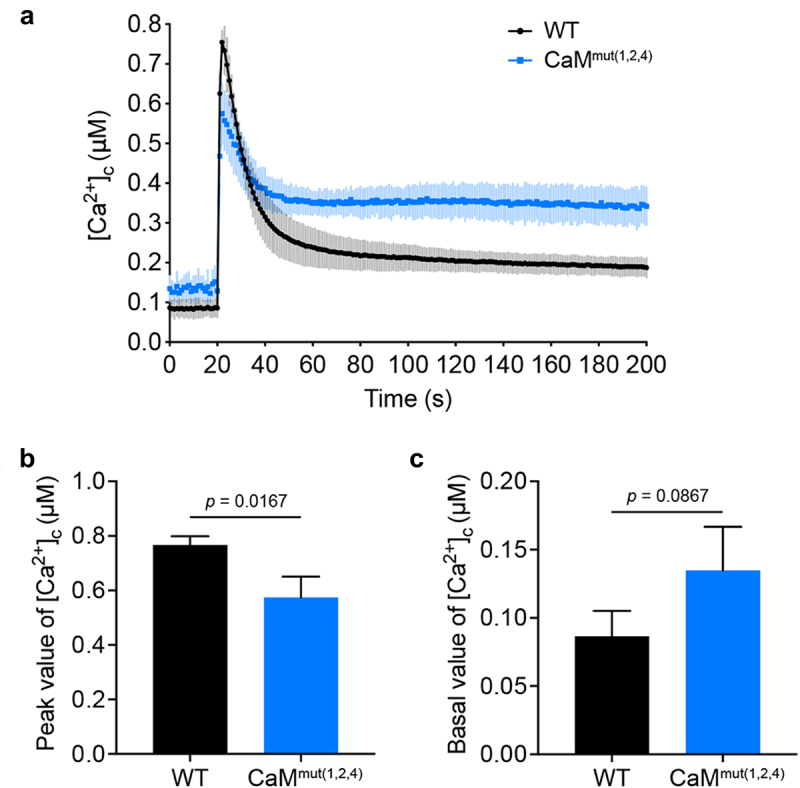
Disorder of cytoplasmic calcium is caused by mutations in Ca**^2+^**-binding sites. (a) linear graphs indicating the real-time [Ca^2+^]_c_ changes in response to calcium stimulus of WT and CaM^mut(1,2,4)^ strains. [Ca^2+^]_c_, the free Ca^2+^ concentration in the cytosol. (b and c) the comparison of [Ca^2+^]_c_ of WT and CaM^mut(1,2,4)^ in dynamic and resting levels. The peak value is the peak level after the extracellular calcium stimulus. The basal level is the resting level prior to the extracellular calcium stimulus. Data are the average of three experiments. Error bars show the standard deviation. Statistical significance was determined by Student’s t-test.

As a well-characterized calcium sensor, CaM regulates cytoplasmic calcium homeostasis by activating various relevant targets, including calcium channels and transporters, in a Ca^2+^-dependent manner [3,12]. However, it is still unknown whether the disruption of Ca^2+^-binding sites influences intracellular calcium homeostasis in *A. fumigatus*. To examine this, we introduced codon-optimized aequorin into WT and CaM^mut(1,2,4)^ strains to monitor extracellular calcium-induced [Ca^2+^]_c_ transients in living cells. The strains that expressed codon-optimized aequorin [39] were constructed by co-transforming the plasmid pAEQ containing aequorin and the selective marker hygromycin B phosphotransferase *hyg* gene into the indicated mutants. CaM^mut(1,2,4)^ was chosen as a representative because it possesses a crucial second calcium-binding loop (CaM2), which allows it to exhibit a defective phenotype. As shown in Figure 6a, when a stimulus was treated with 100 mM CaCl_2_, the [Ca^2+^]_c_ concentration in WT transiently increased from a resting level of approximately 0.07 μM to a peak of 0.74 μM. In comparison, the CaM^mut(1,2,4)^ mutant showed a reduction of 42 ± 4% in the [Ca^2+^]_c_ amplitude under the same stimulating conditions (Figure 6b), implying that mutated calcium-binding sites of CaM reduced extracellular calcium entry and also decreased calcium release from intracellular calcium stores to the cytosol. In contrast, the [Ca^2+^]_c_ basal resting level in the CaM^mut(1,2,4)^ mutant could not return to the normal basal level but instead showed a higher level of intracellular calcium concentration compared to that of WT (Figure 6c), suggesting that calcium binding of CaM affected calcium transport from intracellular to calcium stores. Thus, mutations in the Ca^2+^-binding site of CaM lead to a disorder of cytoplasmic calcium homeostasis.

### Ca^2+^-binding to CaM deficiency causes attenuated virulence in *A.* *fumigatus*

**Figure 7.**
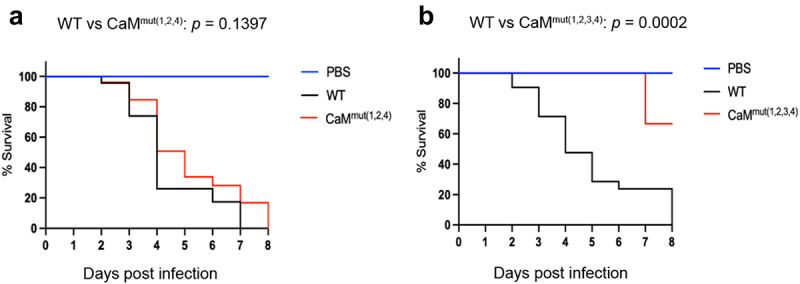
The CaM mutant exhibits attenuated virulence. (a and b) survival curves of *G. mellonella* larvae infected with WT, CaM^mut(1,2,4)^, and CaM^mut(1,2,3,4)^ strains. PBS-injected larvae were used as the negative control. Statistical differences between groups were determined using a log-rank test.

Given that mutations in Ca^2+^-binding sites of CaM resulted in defective phenotypes that are relevant to pathogenesis, such as reduced hyphal growth and disordered cytoplasmic calcium homeostasis, we wondered whether the loss of Ca^2+^-binding sites affects fungal virulence in *A. fumigatus*. To this end, we compared the virulence potential of the WT, CaM^mut(1,2,4)^ and CaM^mut(1,2,3,4)^ strains using a *Galleria mellonella* wax moth model. *G. mellonella* larvae were injected with conidia of the indicated strains and incubated at 37 °C for eight days. The reason for choosing these two mutants was that strains carrying the individual or double mutations did not show any detectable differences compared to WT at 37 °C, suggesting that they are not involved in fungal virulence. Simultaneous mutations in three or four residues resulted in seriously defective phenotypes in *A. fumigatus*. As shown in Figure 7a, CaM^mut(1,2,4)^ exhibited lower mortality than WT, although statistical analysis showed no significant survival difference between WT and CaM^mut(1,2,4)^. However, the survival curve analysis showed that the CaM^mut(1,2,3,4)^ mutant was significantly less virulent than the parental WT (Figure 7b). The percentage of surviving larvae eight days after injection with the CaM^mut(1,2,3,4)^ mutant was 75%. No mortality was observed in the PBS-injected group (Figure 7a and b). These data suggest that simultaneous mutation of the four Ca^2+^-binding sites causes loss of virulence of *A. fumigatus* in the *G. mellonella* insect model.

## Discussion

CaM is a small, ubiquitous Ca^2+^-binding protein that regulates a wide variety of proteins and processes in eukaryotes [38]. In this study, we characterized the role of an evolutionarily conserved CaM protein in the pathogenic fungus *A. fumigatus*. By establishing the conditional *niiA-Afcam* mutant, we demonstrated that turning off *Afcam* expression almost completely blocked hyphal growth and conidiation in *A. fumigatus* (Figure 1a). This suggests that *Af*CaM is important and might be essential, which is consistent with our previous findings in the model filamentous fungus *A. nidulans* [40]. In fact, CaM from vertebrates and *S. pombe* (but not from budding yeast *S. cerevisiae*) can complement each other, indicating that they are relatively functionally conserved, but different species have varied characterization [41–43]. Although bioinformatic analysis indicates that CaM contains four predicted Ca^2+^-binding motifs known as EF-hands that can bind one Ca^2+^ ion each [1], *in vivo* examination of Ca^2+^-binding at individual sites has not been explored before this study in pathogenic fungi. Thus, for an essential gene, the conditional CaM mutants constructed in this study could be used to explore multiple distinct functions of *Af*CaM. Interestingly, strains expressing the individual CaM mutation did not affect hyphal growth and conidiation in *A. fumigatus*, whereas the double mutations exhibited slight growth defects. However, simultaneous mutations of three or four residues resulted in significantly decreased conidia production and colony diameter, suggesting that these four combined Ca^2+^-binding sites of CaM are essential for *A. fumigatus* growth and may have complementary and overlapping roles for Ca^2+^ binding. This finding is consistent with that of *S. pombe* [6]. Notably, the simultaneous mutations of residues 1, 2, 3, and 4 in *Af*CaM resulted in nearly no growth (if any) of *A. fumigatus* colonies (Figure 2d). This is different from that in yeast, in which Ca^2+^-binding defective-CaM mutants only showed decreased growth [10]. In addition, the intracellular localization of GFP-CaM was highly concentrated at the hyphal apex (Figure 3b), indicating that active growth was dependent on the proper trafficking of CaM at the hyphal tip, which explains why *Af*CaM is essential for colony growth. This localization pattern for *Af*CaM is also in line with a previous report on *A. nidulans* [40]. Instead, four Ca^2+^ binding site mutations in *Af*CaM resulted in a diffuse distribution pattern. Nevertheless, *in vitro* biochemistry western blotting further showed that Ca^2+^-binding sites in *Af*CaM affected its protein stabilization. Collectively, these data clearly demonstrate that Ca^2+^-binding sites in *Af*CaM are required for the full functionality of this protein.

As successful environmental fungal pathogens, they must have a strong ability to adapt to various environmental stresses for survival. Accordingly, human pathogenic fungi should face a relative stress barrage in the environment and inside the host body, most notably the requirement for growth at *in vivo* temperatures. Previous studies on the human pathogens *C. neoformans* and *Candida albicans* revealed that the Ca^2+^/CaM-activated calcineurin-dependent pathway is required for adaptation to the stress response and that they are relatively conserved; however, the specific roles of CaM in these fungi have diverged [21,28]. The data in this study (Figure 4) showed that the Ca^2+^ binding mutations in *Af*CaM were hypersensitive to high temperatures, especially when residue 2 was mutated, suggesting that these mutated residues are required for the growth of fungal cells at high temperatures. Interestingly, the defective phenotypes of CaM mutants at 42 °C can be partially restored by high osmotic stress (Fig. S3). This indicates that osmotic pressure can compensate for the low extracellular calcium absorption caused by *Af*CaM Ca^2+^-binding site mutations to maintain calcium homeostasis.

It is well known that CaM plays a crucial role in regulating cellular responses to environmental changes, primarily by activating the CaM-dependent calcineurin-CrzA system, and Ca^2+^ is required for CaM activation [44,45]. The response of *Aspergilli* to elevated concentrations of extracellular calcium is mediated by CrzA [13]. However, nucleocytoplasmic trafficking of CrzA did not occur in Ca^2+^-binding site mutations in CaM mutants (Figure 5), indicating that these Ca^2+^-binding sites are required for the translocation of CrzA from the cytoplasm to the nucleus in *A. fumigatus*. Therefore, the hypersensitivity to high temperatures of *Af*CaM mutants might be due to nuclear mislocalization of CrzA induced by the absence of calcium binding of CaM. Moreover, in previous studies, multiple temperature-sensitive CaM mutants exhibited defects in actin organization, CaM localization, nuclear division, or bud formation [46,47], implying that loss of calcium binding in CaM could cause multiple cellular defects. Importantly, we also demonstrated that the four predicted Ca^2+^-binding sites of CaM are critical for virulence in the wax moth model, implying that the calcium-CaM signalling pathway plays a key role in fungal virulence.

In conclusion, the present study reveals the critical roles of calcium binding in *Af*CaM in regulating hyphal growth, subcellular localization, response to environmental stress, intracellular calcium homeostasis, and virulence in the human pathogen *A. fumigatus*. These findings can serve as a foundation for understanding CaM function in human pathogenic fungi.

## Materials and methods

### Strains and cultivation conditions

The *A. fumigatus* strains used in the present study are listed in the S1 Table. Strains were grown on MM containing 10 g/L glucose, 1 ml/L 1000 × trace elements (2.2 g/L ZnSO_4_·7 H_2_O, 1.1 g/L H_3_BO_3_, 0.5 g/L MnCl_2_·4 H_2_O, 0.5 g/L FeSO_4_·7 H_2_O, 0.16 g/L CoCl_2_·5 H_2_O, 0.16 g/L CuSO4, 0.11 g/L (NH4)_6_Mo_7_O_24_·4 H_2_O, 5 g/L EDTA, pH 6.5–6.8), 50 ml/L 20 × salt solution (60 g/L NaNO_3_, 5.2 g/L KCL, 15.2 g/L KH_2_PO_4_, 5.2 g/L MgSO_4_·7 H_2_O), and 20 g/L agar. MM was supplemented with 5 mM uridine and 10 mM uracil for the uridine and uracil auxotrophic strains. To induce and repress *cam* expression in the *niiA-Afcam* mutant, the medium was supplemented with a nitrogen source of NO_3_^−^ or NH_4_^+^. All strains were cultured at 37 °C or 42 °C.

### Construction of the conditional strain *niiA-Afcam* and the complementary strain *Afcam*-recon

The fusion PCR method was used to generate the indicated mutant strains as previously described [48]. For *niiA-Afcam* mutant construction, the endogenous promoter of *cam* was replaced with a conditional nitrogen-inducible *niiA* promoter. Briefly, approximately 1 kb of the upstream and downstream flanking sequences of the *cam* promoter regions at positions −802 and + 1 were amplified with the primer pairs NA-P1/NA-P3 and NA-P4/NA-P6, respectively. Primers Pyr4-F/Pyr4-R and NA-F/NA-R were used to create the nutritional markers UU and *niiA* promoter fragments, respectively. These two fragments were fused using the primer pair Pyr4-F/NA-R to yield a *cam* deletion cassette. The three purified PCR products were used as templates to generate the *niiA-Afcam* cassette using primers NA-P1 and NA-P6. The resulting fusion product was cloned into the pEASY-Blunt Zero cloning kit (TransGen Biotech) and used to transform the WT recipient strain. Transformants were grown in media supplemented with 0.1 μg/ml pyrithiamine (Sigma) and verified by diagnostic PCR using the primer pairs NA-P1/F-Pyr4, R-Pyr4/NA-P6, and NA(S)/NA(A).

Reintroduction of a full-length *cam* gene into the *niiA-Afcam* mutant strain, including its native promoter, 5’-UTR, ORF, and 3’-UTR, allowing for genetic complementation. With the primers RE-F/RE-R, the *cam* gene fragment was amplified from WT *A. fumigatus* genomic DNA and then fused to a resistance marker, hygromycin B phosphotransferase (*hyg*), using the primers Hyg-F and Hyg-R. The *hyg* gene was amplified from the plasmid pAN7–1 using primers Hyg-F and Hyg-R. The resultant DNA fragment was fused with *hyg* and subsequently transferred to the conditional *niiA-Afcam* strain when cultured on repressing conditions to generate the complementary strain *Afcam*-recon.

### Construction of the mutant *niiA-Afcam^Afcam-T^*

To construct the truncated strain lacking all four EF-loops of *Af*CaM, we used the WT *A. fumigatus* genomic DNA as the template to amplify two 1708 bp and 3468 bp DNA fragments with the primer pairs CaM-T-up/CaM-T1-R and CaM-T1-F/CaM-T-down, respectively. The two PCR products were fused to generate a 5176 bp DNA fragment lacking the first *Af*CaM EF-loop. Next, we used this 5176 bp DNA fragment as the template to amplify two 1780 bp and 3299 bp DNA fragments with the primer pairs CaM-T-up/CaM-T2-R and CaM-T2-F/CaM-T-down, respectively. We then fused these two PCR products to generate a 5079 bp DNA fragment lacking the first and second EF-loops of *Af*CaM. A similar strategy was used to construct strains by deleting all four EF loops. The resultant DNA fragment was subsequently transferred to the conditional *niiA-Afcam* strain to generate the truncated mutant *niiA-Afcam*^*Afcam-T*^ strain.

### Construction of the CaM point mutation strains

A *cam* mutant carrying a single mutation in the first EF-loop (E32A) was constructed as follows. The *pyr4* selectable marker was amplified from plasmid pAL5 with the primer pair Pyr4-F/Pyr4-R. Next, the 5’ flanking sequence (1727 bp) and the 3’ flanking sequence (1653 bp) were amplified from the WT *A. fumigatus* genomic DNA using the primer pairs CaM-mutant-P1/CaM-mutant-P3 and CaM-mutant-P4/CaM-mutant-P6, respectively. The three PCR products were fused to generate a 5502 bp DNA fragment with the primers CaM-mutant-P2/CaM-mutant-P5, which was then cloned into the vector Blunt-Zero to generate the plasmid Pfr01. The 5’ flanking sequence (970 bp) and 3’ flanking sequence (4542 bp) were amplified from plasmid Pfr01 using the primer pairs CaM-mutant-P2/CaM(1)-DOWN and CaM(1)-UP/CaM-mutant-P5, respectively, and the complementary regions contained the desired mutation (glutamate32–alanine32). Using these two fragments as templates, the final 5502 bp fragment was generated by fusion PCR using primers CaM-mutant-P2 and CaM-mutant-P5, which was further cloned into the vector Blunt-Zero to generate plasmid Pfr02. We generated CaM^mut(1)^ by transforming Pfr02 into the WT strain. A similar strategy was used to construct other CaM point mutation strains.

### Construction of the recombinant GFP-CaM strains

To express the N-terminal of the recombinant *niiA-Afcam*^GFP-^^CaM^, we first amplified a 752-bp GFP fragment from plasmid pFNO3 with the primer pair GFP-F/GFP-R. The 5’ flanking sequence (1415 bp) and 3’ flanking sequence (3916 bp) were amplified from the above revertant plasmid using the primer pairs CaM-Q-F/CaM-Q-R and CaM-F/hyg(R), respectively. The three PCR products were fused using primers CaM-P2/hyg(R)-P2 to generate a fusion fragment (5783 bp), which was then transformed into the conditional strain *niiA-Afcam* to generate *niiA-Afcam*^GFP-^^CaM^. To construct a *cam* mutant containing four residue-mutated sites with GFP, the mutated *cam* ORF was amplified from the CaM^mut(1,2,3,4)^ plasmid with the primers CaM-F/CaM-R. The purified PCR product was fused to the 3’-flanking sequence that was amplified from a revertant plasmid using the primers hyg(F)/hyg(R). The resulting fragments were fused to generate the *niiA-Afcam*^GFP-^^CaM^^(1,2,3,4)^ mutant following the same steps as *niiA-Afcam*^GFP-^^CaM^.

### Construction of the CrzA-GFP strains

To express the C-terminus of CrzA-GFP strains, the GFP-*hyg* fragment was first amplified from plasmid pFNO3 using the primers gfp+hyg-UP/gfp+hyg-DOWN. The 5’ flanking sequence (1619 bp) and 3’ flanking sequence (1751 bp) were then amplified from the WT *A. fumigatus* genomic DNA with the primer pairs CRZ-GFP-P1/CRZ-GFP-P3 and CRZ-GFP-P4/CRZ-GFP-P6, respectively. The three PCR products were fused with the primers CRZ-GFP-P2/CRZ-GFP-P5, and then the purified PCR products were transformed into WT, CaM^mut(1,2,4)^ and CaM^mut(1,3,4)^ strains, respectively.

### Construction of the aequorin-expressing strains

To generate aequorin-expressing mutants, the plasmid pAEQS1–15 containing codon-optimized aequorin and the selective marker *hyg* were co-transformed into the indicated mutants. Transformants were screened for aequorin expression using described previously [49].

### Semi-quantitative PCR and RT-qPCR

Fresh *A. fumigatus* conidia were grown in liquid MM in a rotary shaker at 220 rpm at 37 °C for 24 h. Total RNA was extracted with the UNlQ-10 Column TRIzol total RNA isolation kit (Sangon Biotech, B511361) according to the manufacturer’s directions. The HiScript II Q RT SuperMix for qPCR kit (Vazyme, R223–01) was used to synthesize cDNA. For semi-quantitative PCR, with the primers Cam-up/Cam-down and Cam-T-up/Cam-T-down, the *cam* gene fragments were amplified from WT and *niiA-Afcam*^*Afcam-T*^ *A. fumigatus* cDNA and genomic DNA, respectively. For RT-qPCR, independent assays were performed with three replicates, and transcript levels were calculated by the comparative threshold cycle (Δ*C*_*T*_) and normalized against the mRNA expression of *cam* in *A. fumigatus*. The 2^−*ΔΔCT*^ method was used to determine the changes in mRNA expression. All the RT-qPCR primers are given in Table S2.

### Fluorescence microscopy

A total of 1 × 10^6^ conidia of the GFP-CaM strain were cultured on glass coverslips (Sangon Biotech) for cell culture in 1 mL liquid MM for 12 h to visualize the localization of the GFP-CaM fusion protein. The medium was removed and the samples were washed thrice with phosphate-buffered saline (PBS). The samples were fixed for 30 min at room temperature with 4% (v/v) formaldehyde and washed three times with PBS. After that, the nuclear dye 4,’ 6-diamidino-2-phenylindole (DAPI) dissolved in PBS was used at a final concentration of 1 μg/mL and incubated for 30 min at room temperature. DAPI solution was then removed and the glass coverslip was washed three times with PBS. A Zeiss Axio Imager A1 microscope (Carl Zeiss) was used to collect all images.

### [Ca^2+^]_c_ measurement

The strains Aeq-WT and Aeq-CaM^mut(1,2,4)^ were grown on MM for 2.5 days to form fresh conidiation. Equal amounts of conidia in 100 μL of liquid media were distributed to each well of a 96-well microtiter plate. Six wells were used in parallel for each treatment. After the incubation at 37 °C for 18 h, the medium was removed from each well and washed twice with PGM (20 mM PIPES (pH 6.7), 1 mM MgCl_2_, 50 mM glucose). For aequorin reconstruction, we incubated mycelia in 100 μL PGM containing 2.5 μM coelenterazine for 4 h at 4 °C in the dark. After reconstruction, the mycelia were washed twice with PGM, and the plate was incubated at room temperature for 1 h. To chelate extracellular Ca^2+^, 1 mM EGTA was added to each well 10 min before stimulus injection. At the end of each experiment, active aequorin was removed by permeabilizing the cells with 20% ethanol in the presence of 3 M CaCl_2_ to examine the total aequorin luminescence of each well. Luminescence was measured using an LB 96P Microlumat Luminometer (Berthold Technologies). The relative light unit [RLU] values were converted into [Ca^2+^]_c_ concentrations using the calibration formula: pCa = 0.332588 (−log k) +5.5593, where k is luminescence (RLU) s^−1^/total luminescence (in RLU) [39].

### Western blotting analysis

To extract proteins from *A. fumigatus* mycelia, conidia from related strains were incubated in liquid-inducing medium and then shaken at 220 rpm on a rotary shaker at 37 °C for 48 h. The mycelium was ground in liquid nitrogen using a mortar and pestle and suspended in ice-cold extraction buffer (50 mM HEPES (pH 7.4), 137 mM KCl, 10% glycerol, 1 mM EDTA, 1 μg/mL pepstatin A, 1 μg/mL leupeptin, and 1 mM PMSF). Equal amounts of proteins (40 μg) per lane were subjected to 10% SDS – PAGE and transferred to PVDF membranes (Immobilon-P, Millipore) in 384 mM glycine, 50 mM Tris (pH 8.4), and 20% methanol at 250 mA for 1.5 h, the membranes were blocked with PBS, 5% milk, and 0.1% Tween 20. Next, the membrane was probed sequentially with 1:3000 dilutions of anti-GFP primary antibody (Sigma) and goat anti-rabbit IgG-horseradish peroxidase secondary antibody (Abclonal, AS014) diluted in PBS, 5% milk, and 0.1% Tween 20. Blots were developed using Clarity ECL Western blotting detection reagents (Bio-Rad), and images were acquired with a Tanon 4200 Chemiluminescence Imaging System.

### Virulence assay

*G. mellonella* larvae were purchased from Tianjin Huiyude Biotechnology Co., Ltd. In the *G. mellonella* model, 10 μL of the standardized conidia suspension in indicated *A. fumigatus* strains were injected into *G. mellonella* larvae (approximately 0.3 g) via the left prolegs. As a control group, the larvae were injected with PBS. All the larvae were incubated for up to 8 days at 37 °C with humidity levels around 29-33 %, and their survival was evaluated every 24 h. The procedure was conducted three times with groups of 20 larvae per sample.

## Supplementary Material

Fig. S3.pdf

Table S1.docx

Fig. S1.pdf

Table S2.docx

Fig. S4.pdf

Figure S2-revised.pdf

## Data Availability

The authors confirm that data supporting the findings of this study are available within the article.
