# Supplementary figures and images for: Synergistic effects of putative Ca2+-binding sites of calmodulin in fungal development, temperature stress and virulence of Aspergillus fumigatus
Source: Virulence. 2023 Dec 12;15(1):2290757. doi: 10.1080/21505594.2023.2290757 (PMC10761034; doi:10.1080/21505594.2023.2290757)

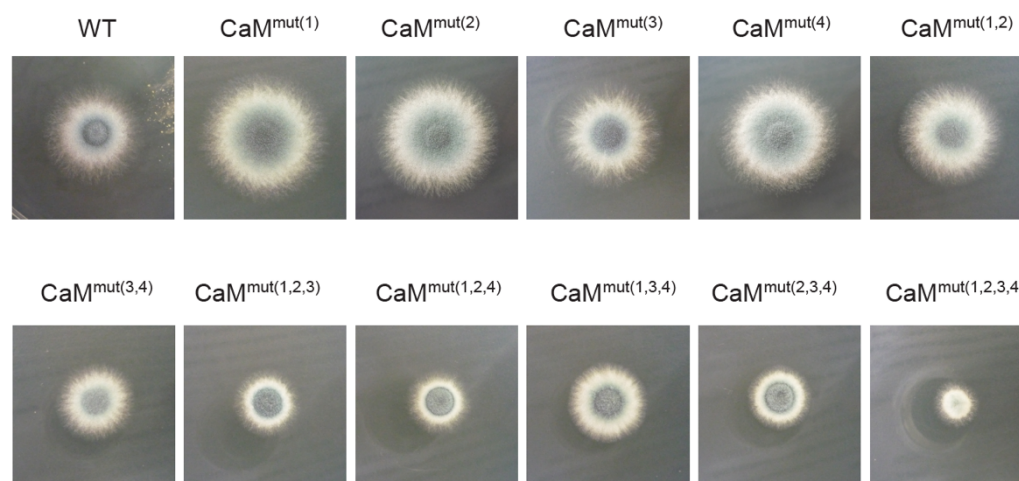

Figure S3: Colony morphology of the indicated *Afcam* mutants under high osmotic stress at 37 °C.

Supplement: Fig. S3.pdf [file KVIR_A_2290757_SM7520.pdf]
