# Supplementary material for: Synergistic effects of putative Ca2+-binding sites of calmodulin in fungal development, temperature stress and virulence of Aspergillus fumigatus
Source: Virulence. 2023 Dec 12;15(1):2290757. doi: 10.1080/21505594.2023.2290757 (PMC10761034; doi:10.1080/21505594.2023.2290757)
Supplement: Table S1.docx [file KVIR_A_2290757_SM7519.docx]

**S1 Table. Strains used in this study**

| **Name** | **Mutated genes** | **Genotype** | **Source** |
| --- | --- | --- | --- |
| *Af*1160 | Parental strain | Δ*KU80 pyrG1* | FGSC |
| FRY1 | CaM(1):: pyrG | Δ*KU80 pyrG1*, Δ*cam*:*cam*^(E32A)^::*pyrG* | This work |
| FRY2 | CaM(2):: pyrG | Δ*KU80 pyrG1*, Δ*cam*::*cam*^(E68A)^::*pyrG* | This work |
| FRY3 | CaM(3):: pyrG | Δ*KU80 pyrG1*, Δ*cam*::*cam*^(E105A)^::*pyrG* | This work |
| FRY4 | CaM(4):: pyrG | Δ*KU80 pyrG1*, Δ*cam*::*cam*^(E141A)^::*pyrG* | This work |
| FRY5 | CaM(12):: pyrG | Δ*KU80 pyrG1*, Δ*cam*::*cam*^(E32A,E68A)^ ::*pyrG* | This work |
| FRY6 | CaM(34):: pyrG | Δ*KU80 pyrG1*, Δ*cam*::*cam*^(E105A,E141A)^::*pyrG* | This work |
| FRY7 | CaM(123):: pyrG | Δ*KU80 pyrG1*, Δ*cam:*:*cam*^(E32A,E68A,E105A)^::*pyrG* | This work |
| FRY8 | CaM(124):: pyrG | Δ*KU80 pyrG1*, Δ*cam*::*cam*^(E32A,E68A,E141A)^:: *pyrG* | This work |
| FRY9 | CaM(134):: pyrG | Δ*KU80 pyrG1*, Δ*cam*::*cam*^(E32A,E105A,E141A)^:: *pyrG* | This work |
| FRY10 | CaM(234):: pyrG | Δ*KU80 pyrG1*, Δ*cam*::*cam*^(E68A,E105A,E141A)^:: *pyrG* | This work |
| FRY11 | CaM(1234):: pyrG | Δ*KU80 pyrG1*, Δ*cam*::*cam*^(E32A,E68A,E105A,E141A)^:: *pyrG* | This work |
| FRY12 | pniiA-CaM | Δ*KU80 pyrG1*, Δ*cam*::*niiA* *(p)*::*cam*::*pyrG* | This work |
| FRY13 | pniiA-CaM^CaM-T^ | Δ*KU80 pyrG1*, Δ*cam*::*niiA* *(p)*::*cam*::*pyrG*; *cam(p)*::*cam(ΔEF-loop)*::*hph* | This work |
| FRY14 | Aeq-CaM(124)-5 | Δ*KU80 pyrG1*, Δ*cam*::*cam* ^(E32A,E68A,E141A)^::*pyrG*; *pAEQS1-15*::*hph* | This work |
| FRY17 | WT^Crz^-GFP | Δ*KU80 pyrG1*, *Δcrz*::*crz*-*GFP*::*hph* | This work |
| FRY18 | CaM(134)^Crz-GFP^ | Δ*KU80 pyrG1*, Δ*cam*::*cam*^(E32A,E105A,E141A)^:: *pyrG* | This work |
| FRY19 | CaM(234)^Crz-GFP^ | Δ*KU80 pyrG1*, Δ*cam*::*cam*^(E68A,E105A,E141A)^::*pyrG* | This work |
| FRY20 | CaM(124)^Crz-GFP^ | Δ*KU80 pyrG1*, Δ*cam*::*cam*^(E32A,E68A,E141A)^::*pyrG* | This work |
