# Supplementary material for: Synergistic effects of putative Ca2+-binding sites of calmodulin in fungal development, temperature stress and virulence of Aspergillus fumigatus
Source: Virulence. 2023 Dec 12;15(1):2290757. doi: 10.1080/21505594.2023.2290757 (PMC10761034; doi:10.1080/21505594.2023.2290757)
Supplement: Fig. S1.pdf [file KVIR_A_2290757_SM7518.pdf]

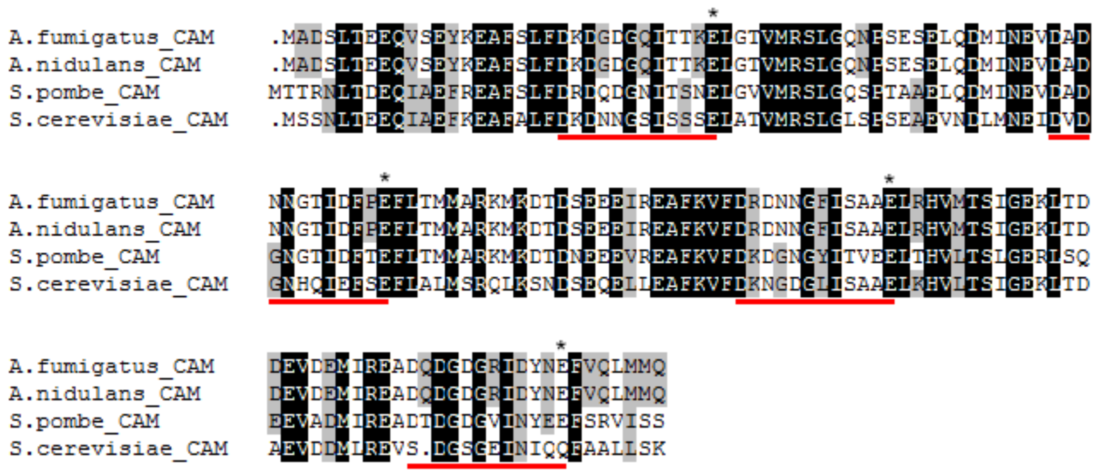


Figure S1: Sequence alignments of CaM homologs in other species. Protein sequence alignment was performed using the Clustal Omega based on the Clustal W multiple sequence alignment method. The four predicted $Ca^{2+}$-binding sites of CaM were highlighted with asterisks. Species names are shown in the figure followed by the GeneBank accession number.
