# Supplementary material for: Synergistic effects of putative Ca2+-binding sites of calmodulin in fungal development, temperature stress and virulence of Aspergillus fumigatus
Source: Virulence. 2023 Dec 12;15(1):2290757. doi: 10.1080/21505594.2023.2290757 (PMC10761034; doi:10.1080/21505594.2023.2290757)
Supplement: Table S2.docx [file KVIR_A_2290757_SM7517.docx]

**S2 Table. Primers used in this study**

| **Name** | **Sequence (5' to 3'）** | **Purpose** |
| --- | --- | --- |
| NA-P1 | GTCACTGTAAATGCAAGG | Fusion PCR for *niiA-Afcam* conditional strain |
| NA-P2 | CATCTGACGACTGAATCG |  |
| NA-P3 | ACACCCGCCAACACCCCGACGAACCTGTACTCCTC |  |
| NA-P4 | GCGTTGAGACTTCGTCACGATGGTATGCATTATTGTTC |  |
| NA-P5 | GACGATGATCCGGATCCAG |  |
| NA-P6 | CGGCAACTGCAGCGCTG |  |
| Pyr4-F | GGGTGTTGGCGGGTGT |  |
| Pyr4-R | TGAATGGCGAATGGCG |  |
| NA-F | CGCCATTCGCCATTCAGCTCAGAGTACTACAGCTG |  |
| NA-R | CGTGACGAAGTCTCAACGC |  |
| F-Pyr4 | GCTGCGTTTCCTGACTTGTC | *niiA-Afcam* conditional strain confirmation |
| R-Pyr4 | TGTCAACATCAAGGGCAC |  |
| NA(S) | AAGGCAACCATAGTCAGG |  |
| NA(A) | AACTCTTTTAGGGGACGG |  |
| RE-F | GGTATGAGCGATTCCTGTG | Complementary *cam* construction |
| RE-R | CGTCCGTCTCTCCGCATGCGGCGTGGCGATTAAAAGAC |  |
| Hyg–F | GCATGCGGAGAGACGGACG |  |
| Hyg–R | TCGAGTGGAGATGTGGAGTGG |  |
| CaM-T1-F | CACGAGATTTGACGTATAG TTGGGCACTGTAATGCGC | Truncated mutant *Afcam*-T construction |
| CaM-T1-R | CTATACGTCAAATCTCGTG |  |
| CaM-T2-F | GATATGATCAACGAGGTG TTCCTTACCATGATGGCTC |  |
| CaM-T2-R | CACCTCGTTGATCATATC |  |
| CaM-T3-F | CGGGAAGCTTTCAAGGTCTTC CTGCGCCACGTTATGACCTC |  |
| CaM-T3-R | GAAGACCTTGAAAGCTTCCCG |  |
| CaM-T4-F | GAGATGATTCGCGAGGCG TTCGTTCAGCTCATGATGC |  |
| CaM-T4-R | CGCCTCGCGAATCATCTC |  |
| CaM-T-up | GGTATGAGCGATTCCTGTG |  |
| CaM-T-down | CGTCCGTCTCTCCGCATGC GGCGTGGCGATTAAAAGAC |  |
| Cam-T-up | GGCCACTGTTCAGCCAGCTTA | Truncated mutant *Afcam*-T confirmation |
| Cam-T-down | CCCGACTAGCTAAGAATGAAC |  |
| CaM-mutant-P1 | CGTCACATCCAGGGTAGAC | Site-directed mutagenesis of *cam* strains and GFP-CaM^mut(1,2,3,4)^ strain construction |
| CaM-mutant-P2 | GTTGCCTTCAAGCCTCAC |  |
| CaM-mutant-P3 | ACACCCGCCAACACCCTTATTTTTGCATCATGAGCTG |  |
| CaM-mutant-P4 | CGCCATTCGCCATTCA GCCTTTCATTTGGCTGTTTTG |  |
| CaM-mutant-P5 | CATACGATGCCTATCACCG |  |
| CaM-mutant-P6 | GAGTGGGCGTGCTTTGATG |  |
| CaM(1)-UP | CACCACCAAGGCATTGGGCACTGTAATGCGCTCTCTGGGC |  |
| CaM(1)-DOWN | GTGCCCAATGCCTTGGTGGTG |  |
| CaM(2)-UP | CTTGTAATCAGCATTCCTTACCATGATGGCTCGGAAGATG |  |
| CaM(2)-DOWN | GGTAAGGAATGCTGATTACAAG |  |
| CaM(3)-UP | CTCCGCTGCGGCGCTGCGCCAC GTTATGACCTCTATCGGG |  |
| CaM(3)-DOWN | GTGGCGCAGCGCCGCAGCGGAG |  |
| CaM(4)-UP | CTAGACAACGCGTTCGTTCAG CTCATGATGCAAAAATAAGC |  |
| CaM(4)-DOWN | CTGAACGAACGCGTTGTCTAG |  |
| GFP-F | GGTATGAGCGATTCCTGTG GGAGCTGGTGCAGGCGCTGG | Fusion PCR for GFP-CaM strain construction |
| GFP-R | CTGAACAATAATGCATACGACCGGTTTATTTGTATAG |  |
| CaM-Q-F | GGTATGAGCGATTCCTGTG |  |
| CaM-Q-R | CATGGTTAAAAATGGGAATG |  |
| CaM-F | GTATGCATTATTGTTCAG |  |
| CaM-R | CGTCCGTCTCTCCGCATGCTTATTTTTGCATCATGAGC |  |
| CRZ-GFP-P1 | CTCAGCATTCCCGTCATGC | Crz-GFP-related strain construction |
| CRZ-GFP-P2 | CAGCAGCATCAAGGGTTTAG |  |
| CRZ-GFP-P3 | CCAGCGCCTGCACCAGCTCCATAGAAGTTACCGGCAGC |  |
| CRZ-GFP-P4 | CACTCCACATCTCCACTCGATGATGAACCATCCGGTCAG |  |
| CRZ-GFP-P5 | CAGTGAGCGGAAGACGAAC |  |
| CRZ-GFP-P6 | CGACACTATGGGCTACGC |  |
| gfp+hyg-UP | GGAGCTGGTGCAGGCGCTGG |  |
| gfp+hyg-DOWN | CACTCCACATCTCCACTCGA |  |
| aeqS-F | ATGACCTCCAAGCAGTAC | Diagnostic PCR for aeqS |
| aeqS-R | TTAGGGGACGGCACCGCCGTA |  |
