# Supplementary material for: Synergistic effects of putative Ca2+-binding sites of calmodulin in fungal development, temperature stress and virulence of Aspergillus fumigatus
Source: Virulence. 2023 Dec 12;15(1):2290757. doi: 10.1080/21505594.2023.2290757 (PMC10761034; doi:10.1080/21505594.2023.2290757)
Supplement: Fig. S4.pdf [file KVIR_A_2290757_SM7516.pdf]

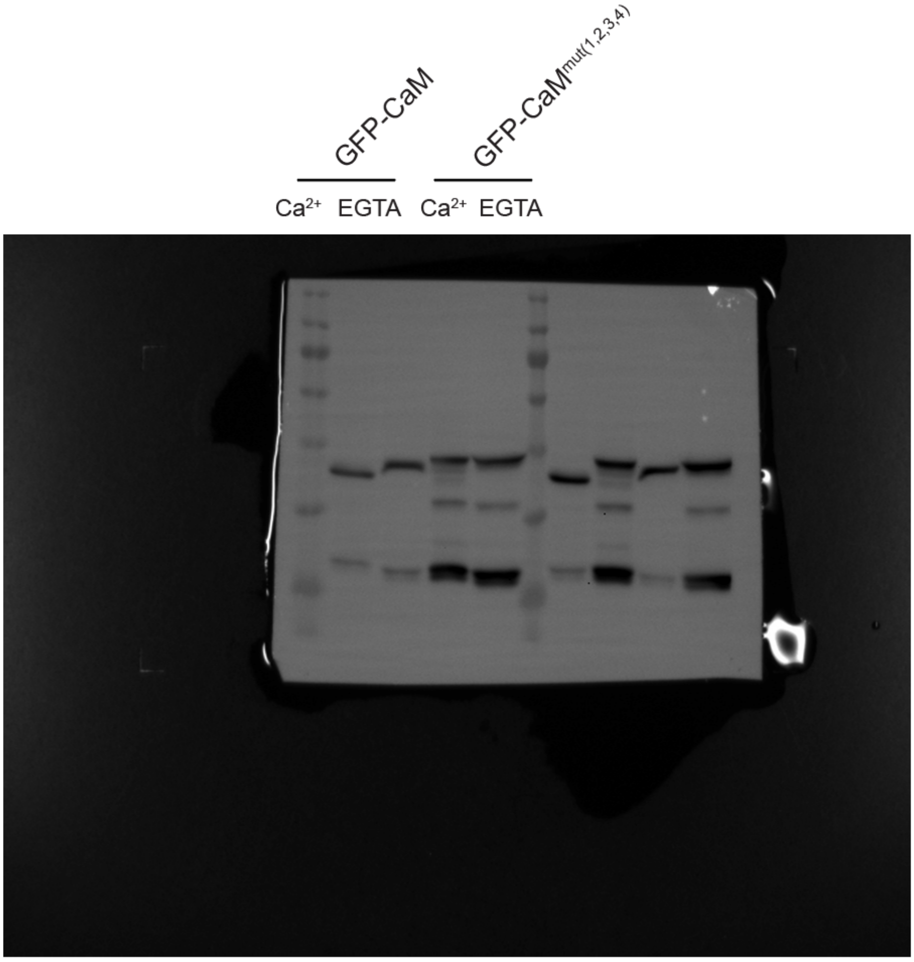

Figure S4: Original image for western blotting anlysis of the distribution of *Af*CaM in the GFP-CaM and GFP-CaM$^{mut(1,2,3,4)}$ strains.
