# Supplementary material for: Synergistic effects of putative Ca2+-binding sites of calmodulin in fungal development, temperature stress and virulence of Aspergillus fumigatus
Source: Virulence. 2023 Dec 12;15(1):2290757. doi: 10.1080/21505594.2023.2290757 (PMC10761034; doi:10.1080/21505594.2023.2290757)
Supplement: Figure S2-revised.pdf [file KVIR_A_2290757_SM7515.pdf]

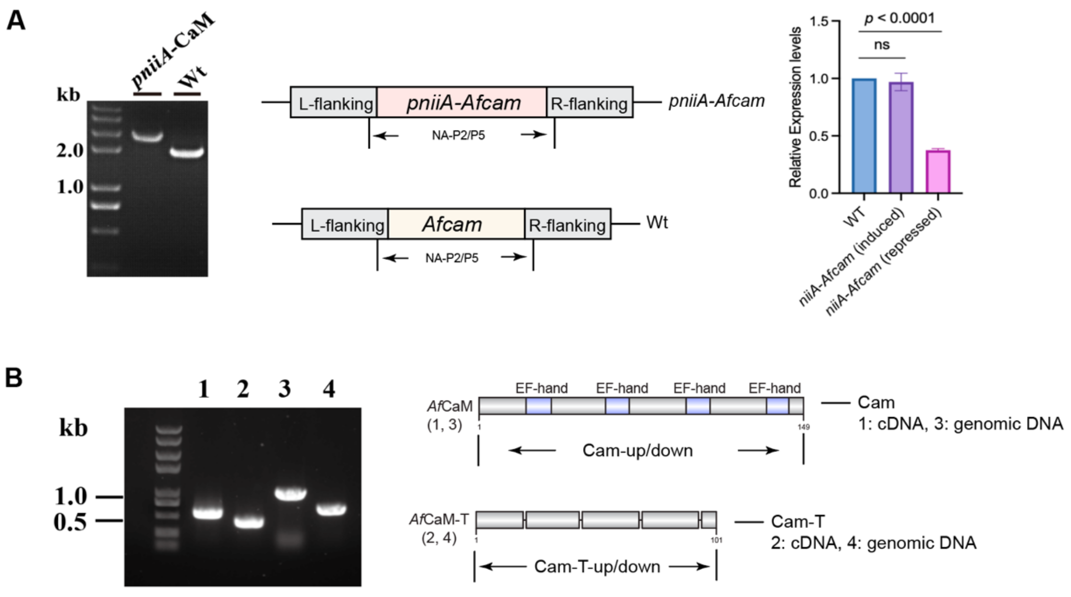

Figure S2. Constructions of *pniiA-Afcam* and *niiA-Afcam*$^{Afcam\text{-}T}$ strains. (A) Diagnostic PCR and RT-qPCR results demonstrated that the conditional *niiA-Afcam* strain was successfully generated. ns, not significant. (B) *niiA-Afcam*$^{Afcam\text{-}T}$ mutant construction was verified by semi-quantitative PCR analysis. 1,2 and 3,4 are fragments which amplified by using WT and *niiA-Afcam*$^{Afcam\text{-}T}$ cDNA and genomic DNA as templates.
